# Oropharyngeal Teratoma: Five-Month-Old Presenting With Failure to Thrive and Severe Obstructive Sleep Apnea

**DOI:** 10.7759/cureus.42578

**Published:** 2023-07-27

**Authors:** Anna Lawrence, Melissa Gener, Shao Jiang, Jill Arganbright

**Affiliations:** 1 Otolaryngology - Head and Neck Surgery, Children's Mercy Hospital, Kansas City, USA; 2 Pathology, Children's Mercy Hospital, Kansas City, USA; 3 Plastic Surgery, Children's Mercy Hospital, Kansas City, USA

**Keywords:** stridor, airway obstruction, congenital abnormality, failure to thrive, obstructive sleep apnea, teratoma

## Abstract

Oropharyngeal teratomas are an extremely rare congenital tumor. They are often diagnosed prenatally and can cause significant airway obstruction and feeding difficulties at birth. We present a five-month-old female that was diagnosed with a palatal teratoma that presented with failure to thrive, difficulty feeding, and eventually with severe obstructive sleep apnea.

We present a five-month-old term, otherwise healthy female who became stridulous after an episode of the respiratory syncytial virus at one month old. At three months old, an otolaryngologist diagnosed mild laryngomalacia with no mass identified, and no surgical intervention was recommended. Due to continued poor weight gain, at four months old, a nasogastric tube was placed. She was subsequently admitted for further workup. She had severe stridor, a failure to thrive, and was in the 0.07th percentile for weight. Workup revealed severe obstructive sleep apnea and a palatal mass obstructing her left oropharynx. A biopsy and debulking of the mass was performed in the operating room. Pathology resulted as a mature teratoma with evidence of glial and intestinal tissue.

There are no pathognomonic characteristics found on imaging to diagnose teratomas, and diagnosis is made with pathologic identification of two of the three germ cell layers. Although most teratomas are benign, there is potential for malignant transformation involving any of the represented germ cell layers. Many teratomas are diagnosed prenatally and can be quite large, often requiring Ex Utero Intrapartum Treatment (EXIT) procedure at birth to establish a safe airway.

Overall, this case highlights the importance of a thorough head and neck exam, including a bilateral flexible laryngoscopy, when evaluating an infant with airway obstruction. Providers evaluating these patients should consider oropharyngeal masses, such as teratoma, as part of the differential to ensure accurate and timely diagnosis.

## Introduction

This article was previously presented as a meeting abstract at the 2022 Children's Mercy Hospital Research Symposium on May 2nd, 2022. Oropharyngeal teratomas are a rare congenital tumor. They are often diagnosed prenatally and can cause significant airway obstruction and feeding difficulties at birth. The patient prognosis is dependent on the respiratory distress of the newborn, as well as the potential risk for malignant transformation and extension of the mass. Teratomas occur in one in 4,000 births and display a female predominance. The head and neck region only represents 5-15% of these tumors, and only 2% are in the oropharynx [1]. There are no pathognomonic characteristics found on imaging; however, calcifications can be suggestive of teratoma [2]. The diagnosis is made by pathologic identification of two of the three germ cell layers [3]. Identification of various tissues such as bone, muscle, exocrine glands, solid organs, intestinal tissue, neuroglial, skin, and teeth is possible within the tumor. Although most teratomas are benign, there is potential for malignant transformation involving any of the represented germ cell layers. Many teratomas are diagnosed prenatally and can be quite large, often requiring Ex Utero Intrapartum Treatment (EXIT) procedure at birth to establish a safe airway [4]. Teratoma diagnosis and treatment are well established in the neonatal population; however, for patients outside of the neonatal age group, it is much less common. Complete surgical excision is considered the gold standard regardless of the presentation age [5]. We present a five-month-old female that was diagnosed with a palatal teratoma that presented with failure to thrive (FTT), difficulty feeding, and severe obstructive sleep apnea (OSA).

## Case presentation

**Figure 1:**
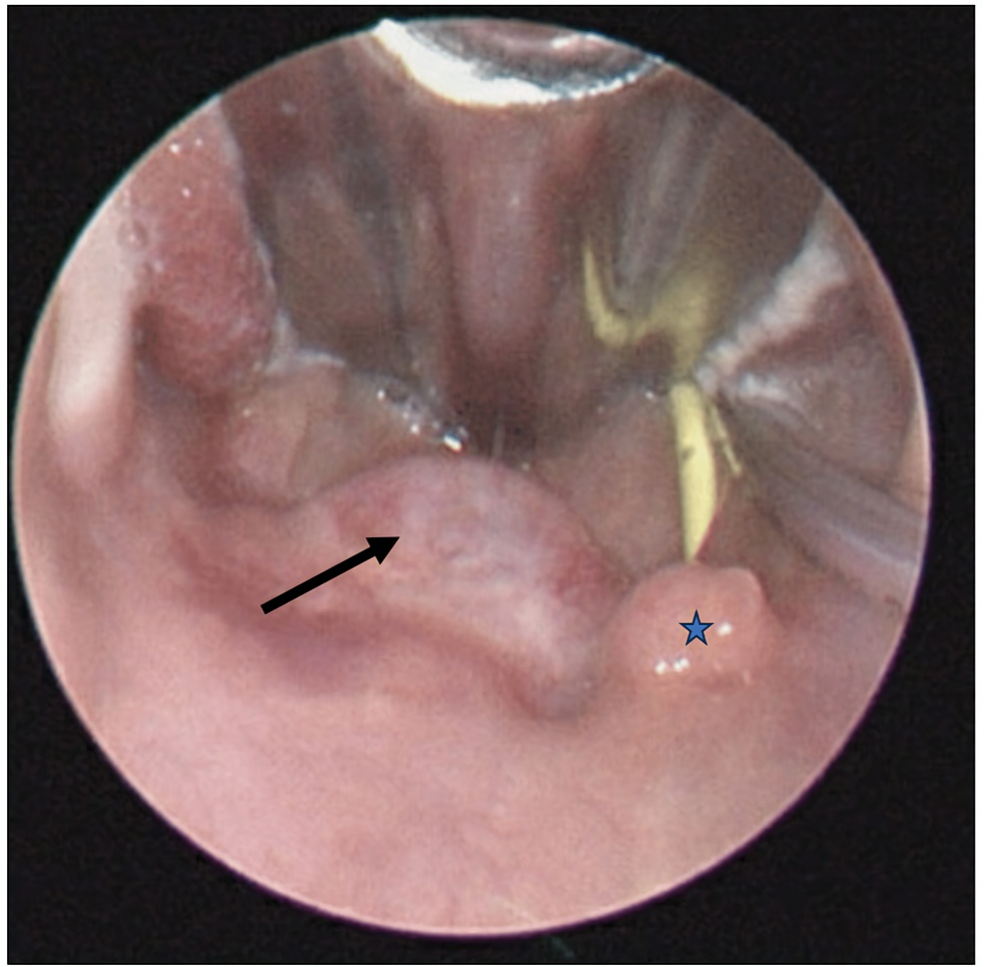
Direct laryngoscopy prior to tumor debulking demonstrating left soft palate submucosal mass Arrow - left soft palate tumor; star - uvula

**Figure 2:**
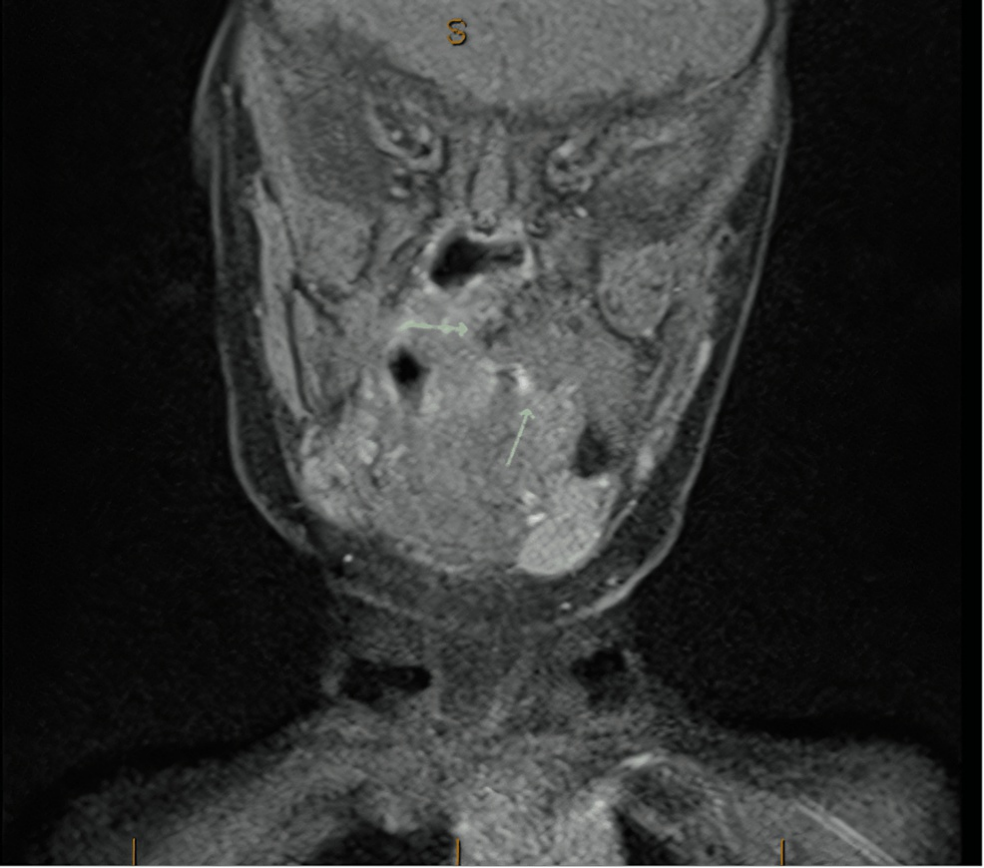
MRI brain T1-weighted post contrast

**Figure 3:**
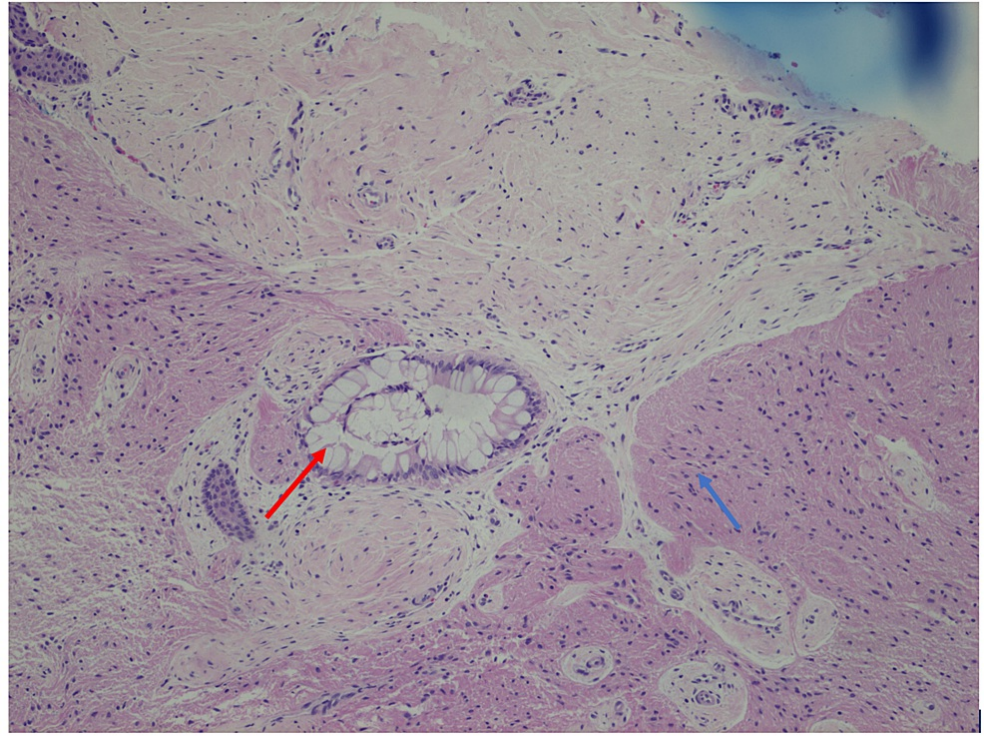
Surgical pathology slides Blue arrow - mature glial tissue; red arrow - intestinal tissue

The patient is a five-month-old term, otherwise healthy female who initially was breastfeeding well. After an episode of respiratory syncytial virus at one month old, she became stridulous and had difficulties with feeding. She was seen by an otolaryngologist at three months of age who performed both an awake and sedated airway evaluation. Findings showed only mild laryngomalacia, and no surgical intervention was recommended. No masses or lesions were visualized at that time. Due to continued poor weight gain, at four months old, a nasogastric tube was placed. Despite enteral feeds, working with a lactation consultant and a feeding specialist, she failed to gain weight, and her breathing became increasingly noisy. She was subsequently admitted to a tertiary care children's hospital for further work-up at age five months. At the time of admission she had severe stridor, FTT, and was in the 0.07th percentile for weight. A sleep study showed severe obstructive sleep apnea with an apnea-hypopnea index of 172.5 events per hour and an oxygen nadir of 74%. Further evaluation by a pediatric otolaryngologist revealed a palatal mass obstructing the entire left oropharynx. Flexible nasopharyngoscopy showed a patent right nasal passage and a large mass obstructing the left nasopharynx. She was taken to the operating room (OR) for a biopsy and debulking of the visible intraoral portion of the mass (Figure 1). A subsequent MRI revealed a 2.2 x 2.2 x 1.8cm expansile left soft palate mass with significant extension into the soft palate musculature (Figure 2). There was no evidence of extension into other surrounding structures. Pathology was signed out as a mature (benign) teratoma with evidence of glial and intestinal tissue (Figure 3).

The patient's status significantly improved with debulking of the tumor. She had a stable airway with reduced obstructive symptoms, her feeding improved, and she was discharged home with close follow-up from otolaryngology and hematology/oncology. Although she did require another operative intervention as part of her treatment, the goal in sending her home was to improve weight gain and nutrition prior to completing a definitive tumor excision.

Seven weeks following her diagnosis, she was then taken to OR for excision of the remaining tumor located within the soft palate. Prior to the final surgical decisions, the medical team and family had a long discussion regarding surgical options, risks, and benefits. An en bloc excision of the left soft palate lesion with margins would ensure complete excision; however, this would require the loss of half the soft palate and would likely create substantial long-term feeding and speech implications. Another alternative discussed was to essentially dissect and peel the tumor out of the soft palate by elevating it off the oral and nasal mucosal layers. This would hopefully offer preservation of the palatal function; however, a more conservative approach for resection could also result in a potentially higher risk for recurrence or persistence of tumor. Ultimately the family and medical team decided to move forward with the palatal preservation surgical approach. The case was performed successfully with all gross tumor removed, and the soft palate was closed primarily without residual defect. Post-operatively she had significant improvement in her breathing, feeding, and sleeping. Her soft palate incisions healed well. She had a three-month post-operative MRI showing post-surgical changes and no residual mass. Since surgery, she has improved her weight-for-age percentile from 0.07th (z-score: -4.10) to 7.10th (z-score: -1.47), gaining 3.82kg. The patient continues to follow closely with otolaryngology and hematology/oncology, with plans for a repeat MRI in four months. If her next MRI shows no residual mass, then we will increase serial imaging increments to six months. 

## Discussion

Oropharyngeal teratomas are extremely rare tumors most often diagnosed in the prenatal/neonatal period. Interestingly, the presented patient was initially seen by a medical professional at three months old, which was the origin of multiple consultations, including lactation, feeding, ear, nose, and throat (ENT), and her pediatrician. Her FTT persisted despite seeing these specialists and undergoing appropriate work-up. FTT is a common problem, representing 5% to 10% of patients seen in the primary care outpatient setting [6]. This results when caloric intake is insufficient to maintain growth. This is a product of either inadequate absorption, inadequate intake, or increased caloric expenditure. There are organic causes, which are reversible with the treatment of underlying disease, and nonorganic causes which tend to improve with behavioral modifications. One does not rule out the other, and both may contribute to a child's poor growth. Approximately 86% of inpatient FTT evaluations are a result of nonorganic etiologies; routine work-up, including laboratory, imaging, and endoscopy studies, reveal a specific organic/anatomical cause less than 1.4% of the time [7]. Although rare to find such a cause of FTT, this case highlights the importance of a complete head and neck exam for patients with this presentation. For an otolaryngology specialist, this includes a thorough oral examination and flexible nasopharyngoscopy bilaterally.

In this case, a bilateral flexible nasopharyngoscopy exam was completed during her evaluation with pediatric otolaryngology. It is unclear if her initial endoscopy by an outside otolaryngologist was completed unilaterally or bilaterally. Interestingly, with the bilateral endoscopy, the initial pass through the right nare did not identify any signs of obstruction. It was not until passing the scope through the left nare that the tumor was seen. This was surprising that such a large lesion could be missed when an endoscopy was performed on the contralateral side, and speaks to the importance of bilateral endoscopy exams. When evaluating a patient for FTT, it is important to rule out airway obstruction. One study found that in pediatric patients with FTT, the prevalence of OSA was 5.5%, compared to the general population, which is between 0.7% and 4.3% [8]. Additionally, patients presenting with FTT and obstructive airway symptoms (as our patient did) are more likely to have an anatomical cause [8]. As stated above, a full exam is important, but this case specifically highlights the importance of scoping bilaterally when seeing infants with FTT and airway obstruction. 

The final treatment plan for this patient resulted from a multidisciplinary discussion of risks and benefits with the family and medical team. One consideration explored was a large palatal resection with mucosal margins and full resection of the tumor. This specific treatment would place her at risk of inability to breastfeed, severe velopharyngeal insufficiency, potential need for speech surgery, and long-standing nasal regurgitation. Given these risks, in addition to the benign pathology, the medical team and family elected for the less invasive approach. To date, no obvious signs of recurrent/residual disease are present; however, she will require close monitoring. Postoperatively, mom was very excited that she was able to resume breast-feeding, and she has not had any evidence of nasal regurgitation. This case demonstrates the need for a personalized decision with caregivers and family, including oncology, surgeon, and family.

## Conclusions

In conclusion, this case highlights the importance of a thorough head and neck exam, including a bilateral flexible laryngoscopy, when evaluating an infant with FTT and airway obstruction. Providers evaluating these patients should consider oropharyngeal masses, such as teratoma, as part of the differential to ensure accurate and timely diagnosis. Treatment for teratomas is surgical excision; however, it should be tailored to the individual patient with a shared decision-making model involving the family and medical team. 
